# Obesity, daily life restrictions, and health behaviors during the COVID-19 pandemic in Korea

**DOI:** 10.3389/fpubh.2025.1653576

**Published:** 2025-10-31

**Authors:** Inwook Lee, Yujin Chang, Hye Soon Park, Jung Ah Lee

**Affiliations:** Department of Family Medicine, Asan Medical Center, University of Ulsan College of Medicine, Seoul, Republic of Korea

**Keywords:** COVID-19 pandemic, social distancing, daily life restrictions, obesity, physical activity, fastfood intake

## Abstract

**Introduction:**

The COVID-19 pandemic led to implementation of social distancing policies, possibly affecting lifestyle changes and restricting daily life. This study aimed to investigate the association between health behaviors or restrictions and obesity.

**Methods:**

This cross-sectional study was conducted with Korean adults (≥19 years) from the 2020 and 2021 Korean Community Health Surveys. Considering the guidelines for Korean obesity population, participants were grouped by body mass index (BMI): underweight (BMI < 18.5 kg/m^2^), normal (BMI 18.5–24.9 kg/m^2^), obesity I (BMI 25–29.9 kg/m^2^), and obesity II (BMI ≥ 30 kg/m^2^). Health behavior changes and daily life restrictions were measured using a self-report questionnaire.

**Results:**

Among men, daily life restrictions increased in obesity I (OR 1.10, 95% CI 1.07–1.13) and obesity II (OR 1.10, 95% CI 1.04–1.17) compared to the normal weight group. Decreased physical activity and increased fast food consumption tended to increase with obesity grade (*p* < 0.001). Among women, decreased physical activity was associated with both obesity I (OR 1.14, 95% CI 1.11–1.17) and obesity II (OR 1.20, 95% CI 1.12–1.28). Increased fast food consumption were also associated with obesity I (OR 1.12, 95% CI 1.07–1.17) and obesity II (OR 1.24, 95% CI 1.13–1.35). Both factors tended to increase with obesity grade (*p* < 0.001).

**Conclusion:**

During the COVID-19 pandemic, the self-reported restrictions on daily life and unhealthy behaviors have progressively increased among people with obesity, depending on the severity of their obesity. Therefore, it is necessary to reinforce obesity prevention and management, particularly in vulnerable populations.

## Introduction

A novel coronavirus, known as coronavirus disease 2019 (COVID-19), was identified in late 2019 and rapidly spread worldwide, becoming the most significant global public health threat of the century (1). To prevent the spread of COVID-19, the Korean government implemented a social distancing policy on March 21, 2020 (2). It included limits on the number of people in gatherings, permitted crowd sizes at sports facilities, requirements for electronic log systems, restrictions on business hours, and bans on eating food in indoor public spaces—all of which can affect individual’s social activities (2).

Obesity is an important risk factor for various chronic diseases, including type 2 diabetes, cardiovascular diseases, musculoskeletal diseases, and several types of cancer (3). According to the 2023 Obesity Fact Sheet of Korea, the prevalence of obesity increased from 30.2% in 2012 to 38.4% in 2021, and that of abdominal obesity increased from 21.4 to 31.0% in the same period of time (4). These consistent upward trend in obesity prevalence has been observed across both sexes and all age groups in Korea (4). Previous studies have reported that the prevalence of obesity, morbid obesity, and mean BMI increased during the COVID-19 pandemic compared to the pre-pandemic period (5–7).

The implementation of social distancing policies during the COVID-19 pandemic may have influenced changes in health behaviors and restrictions on daily life for the general population. Additionally, these unhealthy lifestyle factors may be associated with social distancing policies, such as a lack of physical activity (8). Therefore, our study aimed to investigate the association between restrictions on daily life during COVID-19 pandemic and obesity levels in Korean population using the Korea Community Health Survey (KCHS).

## Methods

### Study design and setting

**Figure 1:**
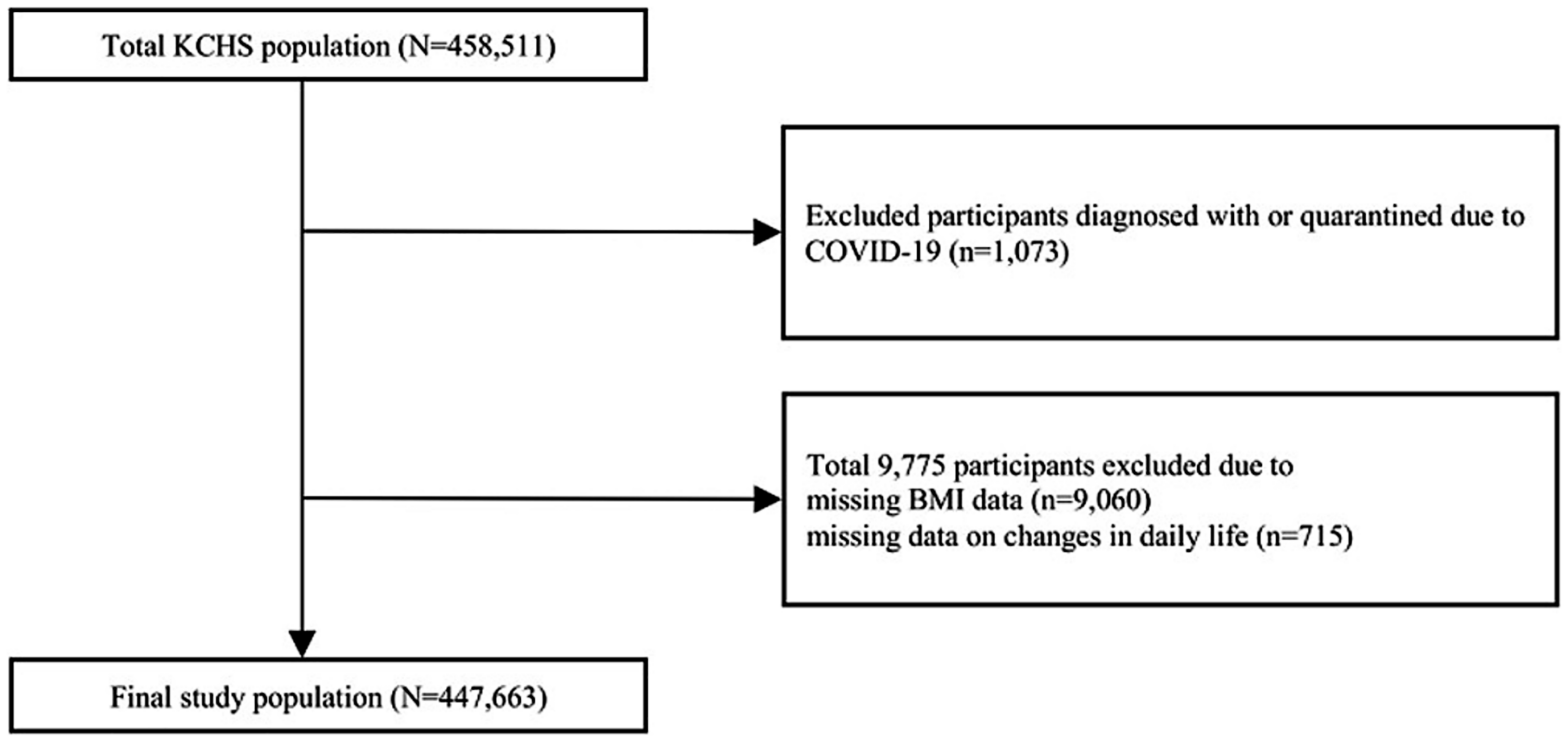
Flow chart of the participants.

The KCHS is a nationwide, community-based survey conducted by the Korea Centers for Disease Control and Prevention to investigate the public health status and health behaviors of individuals aged 19 years or older (9). It covers the provinces of 254 districts across 17 metropolitan cities, with household systematically selected based on local districts. Approximately 900 individuals from each district were chosen through a multistage probability sampling design based on community classification and type of residence (9). A total of 458,511 participants were surveyed in 2020 and 2021. We excluded 10,848 individuals who were either diagnosed with or quarantined due to COVID-19 (*n* = 1,073), had missing BMI data (*n* = 9,060), or had missing data on daily life changes during the COVID-19 pandemic (*n* = 715). Finally, 447,663 individuals were included in the analysis (Figure 1). This study met the criteria for exemption from the Institutional Review Board because of the use of anonymized KCHS data open to the public.

Basic characteristics were investigated including age, sex, educational level, income, and marital status. Educational level was categorized as less than high school (<12 years), high school graduate (12 years), and more than high school (>12 years). Income was categorized as follows: low (<2,000,000 KRW [1 USD = 1,233 KRW]), middle (2,000,000–3,999,999 KRW), and high (≥4,000,000 KRW). Marital status was categorized as married/cohabited, separated/widowed/divorced, or never-married single. Occupational status was categorized based on the International Standard Classification of Occupations (ISCO) into the following groups; ① Managers, Professionals, ② Clerical Support workers, ③ Service, Sales workers, ④ Agricultural, Forestry, Fishery workers, ⑤ Craft, Machine operators, ⑥ Elementary occupations, ⑦ Armed Forces occupations, ⑧ Unemployed, Student, Housemakers.

Body mass index (BMI) was calculated as weight in kilograms divided by height in meters squared (kg/m^2^), based on self-reported height and weight. According to the guidelines for the management of obesity by the Korean Society for the Study of Obesity, the cutoff levels for the definition of overweight and obesity were BMI ≥ 23 kg/m^2^ and ≥25 kg/m^2^, respectively (10). Considering these definitions, we divided the participants into four groups: underweight (BMI < 18.5 kg/m^2^), normal (BMI ≥ 18.5 kg/m^2^ and <25 kg/m^2^), obesity I (BMI ≥ 25 kg/m^2^ and <30 kg/m^2^), and obesity II (BMI ≥ 30 kg/m^2^) groups.

### Assessment of daily life changes during the COVID-19 pandemic

Daily life changes were evaluated using the following question: “Assume that daily life before the COVID-19 outbreak is 100 points. What score would you give in your current daily life compared to before the COVID-19 outbreak? A score of zero indicates complete restriction on daily life. A smaller score indicates a greater change in daily life restrictions. The perfect score would be 100, if you do not feel any additional restriction at the current moment during the COVID-19 pandemic.” We divided the response scores into quartiles, with the first quartile (0–40) was considered to indicate the presence of daily life restriction. In addition, changes in specific aspects of daily life were assessed such as physical activity and fast-food consumption. For each category, individuals could answer whether their activity increased, decreased, or remained unchanged compared to the period before the COVID-19 pandemic.

### Statistical analysis

Since the Korea Community Health Survey is a sample survey rather than a complete enumeration, the analysis was conducted using a complex sample design to reduce bias when estimating results for the general population. The stratification variable was *kstrata* (considering region, age, and sex), the cluster variable was *spot_no* (representing sampling are number), and the sampling weight variable was *wt_p*. The characteristics of the study population are presented as unweighted sample counts and weighted percentages stratified by sex. We compared the presence of daily life restrictions, decreased physical activity, and increased fast food consumption according to obesity groups, which are presented as weight percentage with *P* for trend. Since the obesity group was categorized into four groups, Bonferroni correction was applied for multiple comparisons, and a *p* value less than 0.0083 was considered statistically significant. *P* for trend was calculated using linear regression based on an ordinal categorical variable of obesity. Logistic regression analyses were performed to calculate the odds ratios (ORs) and 95% confidence intervals (CIs) for the presence of daily life restrictions according to the BMI group, with the BMI group treated as a categorical variable. In logistic regression analysis, *P* for trend was calculated based on an ordinal categorical variable of obesity. Multivariable analysis was performed after adjusting for age, sex, marital status, income, educational status, and occupational status. Low income and low educational status are well-known risk factors of obesity (11, 12), and marital status has also influenced body weight (13). Additionally, occupational types are closely associated with obesity (14). Therefore, we adjusted for these variables as potential confounders in our analysis. We conducted a complete case analysis, as missing values accounted for less than 5% of the data. Sensitivity analysis was performed using score-based cut-offs, dividing individuals whose daily life was restricted to 50 points or less into intervals of 10 points. Since a score of 100 indicated no restriction, a score of half or lower was regarded as experiencing restriction. All statistical analyses, including survey (svy) analysis, were performed using STATA 16.1 (Stata Corporation, College Station, Texas, USA). Except for multiple comparisons, a two-tailed *p* < 0.05 was considered statistically significant.

## Results

### Basic characteristics of study participants

Among the 447,663 study participants, 206,299 (46.08%) were men and 241,364 (53.92%) were women, with an average age of 48.0 years in men and 49.66 years in women (Table 1). According to the BMI categories, 34.00% of men and 18.90% of women were in the obesity I group. Meanwhile 5.78% men and 3.02% women were classified into the obesity II group. A total of 25.49% of men and 31.22% of women reported having subjective daily life restrictions.

**Table 1:** Basic characteristics of study participants.

Variables	Total (*N* = 447,663)	Men (*N* = 206,299)	Women (*N* = 241,364)
	Mean ± SE^a^ or unweighted *N* (weighted %)		
Age (years)			
Mean (SE)	48.82 ± 0.04	48.0 ± 0.05	49.66 ± 0.05
<40	101,160 (32.70)	49,155 (34.21)	52,045 (31.20)
40–59	158,040 (39.00)	74,013 (39.73)	84,027 (38.28)
≥60	188,463 (28.30)	83,171 (26.06)	105,292 (30.52)
Education			
>High school	175,076 (51.85)	91,577 (56.64)	83,499 (47.09)
High school	129,983 (28.53)	62,804 (28.85)	64,179 (28.22)
<High school	145,239 (19.54)	51,744 (14.44)	93,495 (24.61)
Unknown	365 (0.08)	174 (0.07)	191 (0.08)
Household income (KRW/month)			
≥4,000,000	175,697 (49.20)	84,386 (50.56)	91,311 (47.84)
2–3,999,999	129,170 (28.71)	62,735 (29.60)	66,435 (27.82)
<2,000,000	134,666 (19.89)	55,420 (17.69)	79,246 (22.07)
Unknown	8,130 (2.21)	3,758 (2.15)	4,371 (2.26)
Occupational status			
Managers, professionals	49,237 (14.77)	25,076 (16.15)	24,161 (13.40)
Clerical support workers	40,694 (12.39)	20,515 (13.51)	20,179 (11.27)
Service, sales workers	57,605 (13.56)	20,673 (11.83)	36,932 (15.29)
Agricultural, forestry, fishery workers	42,778 (2.82)	24,875 (3.58)	17,903 (2.06)
Craft, machine operators	40,903 (10.68)	36,254 (19.28)	4,649 (2.14)
Elementary occupations	45,223 (8.49)	20,093 (8.82)	25,130 (8.16)
Armed forces occupations	1,390 (0.27)	1,307 (0.51)	83 (0.03)
Unemployed, student	169,572 (36.97)	57,383 (26.26)	112,189 (47.6)
Unknown	261 (0.05)	123 (0.06)	138 (0.05)
Self-reported BMI			
Mean (SE)	23.65 ± 0.01	24.53 ± 0.01	22.77 ± 0.01
<18.5 kg/m^2^	18,969 (4.27)	4,473 (1.82)	14,496 (6.70)
18.5–24.9 kg/m^2^	292,742 (64.91)	124,172 (58.40)	168,570 (71.38)
25–29.9 kg/m^2^	118,254 (26.42)	67,528 (34.00)	50,726 (18.90)
≥30 kg/m^2^	17,698 (4.40)	10,126 (5.78)	7,572 (3.02)
Presence of daily life restriction			
No	330,131 (71.63)	156,444 (74.51)	173,687 (68.78)
Yes	117,532 (28.37)	49,855 (25.49)	67,677 (31.22)

a The analysis was performed using complex survey sampling weights.

### Changes in health behaviors during COVID-19 pandemic according to obesity

Table 2 presents the changes in health behaviors during the COVID-19 pandemic according to obesity status based on BMI. In men, daily life restrictions increased in 24.70% of the normal weight group, 26.54% of the obesity I group, and 27.29% of the obesity II group. The prevalence of daily life restrictions increased with a higher degree of obesity (*P* for trend < 0.001). Compared with the normal weight group, men in the obesity I and obesity II groups showed approximately 1.84 and 2.59% higher prevalence of daily life restrictions, respectively. No association was observed between the presence of daily life restrictions and obesity classified by BMI (*p* = 0.324) in women.

**Table 2:** Changes in health behaviors during COVID-19 pandemic according to obesity status.

Variables	Underweight (BMI < 18.5 kg/m^2^)	Normal (BMI 18.5–24.9 kg/m^2^)	Obesity I (BMI 25–29.9 kg/m^2^)	Obesity II (≥30 kg/m^2^)	*P* value^b^	*P* for trend^c^
	Unweighted *N* (weighted %)					
Men						
Presence of daily life restriction	1,038 (25.74)	28,989 (24.70)	17,092 (26.54)	2,736 (27.29)	0.001	<0.001
95% CI^a^	23.96–27.61	24.36–25.04	26.10–27.00	26.18–28.44		
Decreased physical activity	1,415 (40.22)	45,235 (43.73)	28,007 (48.66)	4,587 (52.28)	<0.001	<0.001
95% CI	38.07–42.41	43.33–44.14	48.14–49.18	50.99–53.57		
Increased fast food consumption	397 (20.45)	13,843 (20.35)	10,353 (24.35)	2,266 (29.50)	<0.001	<0.001
95% CI	18.38–22.70	19.97–20.73	23.86–24.86	28.28–30.74		
Women						
Presence of daily life restriction	4,028 (31.68)	47,154 (31.20)	14,278 (31.14)	2,217 (31.08)	0.324	0.138
95% CI	30.65–32.73	30.88–31.53	30.57–31.72	29.68–32.51		
Decreased physical activity	5,976 (50.76)	74,427 (52.91)	22,610 (53.89)	3,512 (56.14)	<0.001	<0.001
95% CI	49.63–51.90	52.56–53.26	53.26–54.51	54.57–57.71		
Increased fast food consumption	2,376 (28.75)	23,995 (27.42)	6,091 (24.73)	1,263 (29.56)	<0.001	<0.001
95% CI	27.62–29.91	27.05–27.80	24.06–25.41	27.91–31.26		

a The analysis was performed using complex survey sampling weights.

b *P* value was calculated using chi-square test. Using the Bonferroni correction, *p* value less than 0.0083 is considered statistically significant for each pairwise comparison.

c *P* for trend was calculated using linear regression based on a categorical variable of obesity.

To examine the specific aspects of daily life restrictions, we investigated changes in physical activity and fast food consumption. Among men, the proportion of those who reported decreased physical activity was 43.73% in the normal weight group, 48.66% in the obesity I group, and 52.28% in the obesity II group. The proportion of those who reported an increase in fast food consumption was 20.35, 24.35, and 29.50% in the normal weight, obesity I, and obesity II groups, respectively. The proportion of men reporting decreased physical activity and increased fast-food consumption rose by 4–5% in the obesity I group and 8–10% in the obesity II group, indicating a graded increase with higher obesity levels.

Among women, the proportion of those who reported decreased physical activity was 52.91% in the normal weight group, 53.89% in the obesity I group, and 56.14% in the obesity II group. The proportion of women with increased fast food consumption was 27.42, 24.73, and 29.56% in the normal weight, obesity I, and obesity II groups, respectively. Obesity classified by BMI was significantly associated with increased fast-food consumption (*p* < 0.001) and decreased physical activity (*p* < 0.001), with trends according to the degree of obesity (*P* for trend < 0.001 for both outcomes). Compared with the normal weight group, women in the obesity I and obesity II groups showed approximately 1–3% higher prevalence of decreased physical activity, while increased fast-food consumption was observed only in the obesity II group, with a 2–3% increase.

### Logistic regression analysis of daily life restriction and changes in health behaviors according to obesity

We analyzed daily life restrictions and changes in health behaviors according to the degree of obesity, stratified by sex (Table 3). In men, both the obesity I (OR = 1.10 95% CI 1.07–1.13) and obesity II groups (OR = 1.10, 95% CI 1.04–1.17) were more likely to report daily life restrictions than the normal BMI group, and this association strengthened with the degree of obesity (*P* for trend < 0.001). In women, obesity was not associated with restrictions in daily life. In men, decreased physical activity was more prevalent among the obesity I (OR = 1.17, 95% CI 1.13–1.20) and the obesity II group (OR = 1.27, 95% CI 1.20–1.34). A significant trend was also observed (*P* for trend < 0.001). In women, decreased physical activity was more frequently observed in both the obesity I (OR = 1.14, 95% CI 1.11–1.17) and obesity II groups (OR = 1.20, 95% CI 1.12–1.28) with a trend of increase according to the degree of obesity (*P* for trend < 0.001). In both men and women, the increased fast food consumption was more prevalent among the obesity I group (OR = 1.20, 95% CI 1.15–1.24 in men and OR = 1.12, 95% CI 1.07–1.17 in women) and the obesity II group (OR = 1.33, 95% CI 1.25–1.42 in men and OR = 1.24, 95% CI 1.13–1.35 in women) compared to the normal group. Additionally, increased fast food consumption during COVID-19 pandemic was positively associated with higher levels of obesity (*P* for trend < 0.001).

**Table 3:** Logistic regression analysis of daily life restrictions and changes in health behaviors according to obesity status.

Variables	Univariable OR (95% CI)	*P* value	*P* for trend^a^	Multivariable OR^b^ (95% CI)	*P* value	*P* for trend^a^
Men						
Daily life restriction			*<0.001*			*<0.001*
Underweight	1.06 (0.96–1.16)	0.262		1.01 (0.91–1.11)	0.891	
Normal	1 (reference)			1 (reference)		
Obesity I	1.10 (1.07–1.33)	<0.001		1.10 (1.07–1.13)	<0.001	
Obesity II	1.14 (1.07–1.21)	<0.001		1.10 (1.04–1.17)	0.002	
Decreased physical activity			*<0.001*			*<0.001*
Underweight	0.87 (0.79–0.95)	0.002		0.93 (0.84–1.02)	0.110	
Normal	1 (reference)			1 (reference)		
Obesity I	1.22 (1.19–1.26)	<0.001		1.17 (1.13–1.20)	<0.001	
Obesity II	1.41 (1.34–1.49)	<0.001		1.27 (1.20–1.34)	<0.001	
Increased fast food consumption			*<0.001*			*<0.001*
Underweight	1.01 (0.88–1.15)	0.925		0.96 (0.84–1.11)	0.591	
Normal	1 (reference)			1 (reference)		
Obesity I	1.26 (1.22–1.31)	<0.001		1.20 (1.15–1.24)	<0.001	
Obesity II	1.64 (1.54–1.74)	<0.001		1.33 (1.25–1.42)	<0.001	
Women						
Daily life restriction			*0.135*			*0.358*
Underweight	1.02 (0.97–1.07)	0.383		0.99 (0.94–1.04)	0.763	
Normal	1 (reference)			1 (reference)		
Obesity I	1.00 (0.97–1.03)	0.843		1.02 (0.99–1.05)	0.110	
Obesity II	0.99 (0.93–1.06)	0.864		0.99 (0.93–1.06)	0.743	
Decreased physical activity			*<0.001*			*<0.001*
Underweight	0.92 (0.88–0.96)	<0.001		0.87 (0.83–0.92)	<0.001	
Normal	1 (reference)			1 (reference)		
Obesity I	1.04 (1.01–1.07)	0.006		1.14 (1.11–1.17)	<0.001	
Obesity II	1.14 (1.07–1.22)	<0.001		1.20 (1.12–1.28)	<0.001	
Increased fast food consumption			*<0.001*			*<0.001*
Underweight	1.07 (1.01–1.13)	0.027		0.86 (0.81–0.91)	<0.001	
Normal	1 (reference)			1 (reference)		
Obesity I	0.87 (0.84–0.91)	<0.001		1.12 (1.07–1.17)	<0.001	
Obesity II	1.11 (1.02–1.21)	0.013		1.24 (1.13–1.35)	<0.001	

a *P* for trend was calculated based on an ordinal categorical variable of obesity.

b Adjusted for age, income, education, marital status, and occupational status.

In the sensitivity analysis, the overall trend in men was consistent, and the association between obesity and daily life restriction remained across cut-offs of 10, 20, 30, 40, and 50 points (Supplementary Table 1). With a cut-off of 10 points, the ORs (95% CI) among men were 1.23 (1.13–1.34) for obesity I and 1.32 (1.13–1.55) for obesity II. When the cut-off was increased to 50 points, the ORs were slightly attenuated to 1.14 (1.10–1.18) for obesity I and 1.12 (1.04–1.20) for obesity II. These results indicate that obesity is associated with approximately a 10–20% higher likelihood of daily life restriction, decreased physical activity, and increased fast-food consumption. In contrast, among women, the associations were not consistently significant across different cut-off values.

## Discussion

In this study, self-reported restrictions on daily life during COVID-19 were more frequently reported by obese men than by those with normal weight. Additionally, both decreased physical activity and increased fast food consumption were more common among the obese group. In women, daily life restrictions did not significantly differ between BMI groups. However, similar to men, decreased physical activity and increased fast-food consumption were more frequent in the obese group. These two factors also showed a significant trend across increasing obesity levels.

This study showed differences in the presence of daily life restrictions, decreased physical activity, or increased fast food consumption based on obesity levels. These unhealthy lifestyle factors were observed during the study period when social distancing policies were implemented in Korea and their prevalence varies according to the degree of obesity. Previous studies have reported that social distancing policies and restrictions on daily activities contribute to increased obesity and weight gain (15, 16). A large study conducted among older adults in England reported that a short 2-month lockdown in 2020 during COVID-19 pandemic led to weight gain, which was associated with decreased physical activity, increased sedentary behavior, increased food intake, and longer sleep duration (15). However, it focused on the health behaviors changes related with weight gain during lockdown and did not compare weight changes between the normal weight and obese group. A study from Brazil similarly found that social distancing policies were related with weight gain (16). Specifically, while 46.6% of individuals with a normal BMI reported weight gain, this proportion rose to over 87% among those classified as overweight or obese. Also, the overweight and obese groups reported less total weekly physical activity and more frequent binge eating compared to the normal-weight group (16). However, the study had a small sample size based on an online survey, which may limit the generalizability of the findings.

We found that individuals with obesity were more likely than those with normal weight to experience limitations in daily life, reduced physical activity, and increased consumption of fast food during the implementation of social distancing policies. Moreover, the finding of this study is that these tendencies became more pronounced with increasing obesity severity. Supporting our findings, a recent qualitative study has shown that obese individuals find more challenging to make healthier choices when faced with the option between a healthier choice and an easier choice (17). Additionally, although the study focused on children, a recent study about Austrian pediatric population reported that approximately half of overweight children became obese during the pandemic (18).

In our study, the presence of self-reported restrictions on daily life increased among obese men, but did not differ by weight group among women. Similar with our study, a previous study about Korean general population showed that obesity increased from 2019 to 2020 among men but not women, which was mainly associated with decreased physical activity (19). In Korea, since men generally tend to have higher levels of physical activity compared to women, it is possible that men’s physical activity may have been more affected by COVID-19 pandemic (20). Additionally, women usually have more social pressure to maintain their weight in Korea (21). A previous study in Korea reported that women engaged in more weight control efforts, which were associated with their subjective perception of their body rather than their actual BMI (22). Thus, women may have paid more attention to weight management than men during the COVID-19 pandemic. From a biological perspective, cortisol levels in men tend to be higher than in women in response to stress (23). Perceived stress may influence HPA axis function, thereby affecting the risk of obesity and metabolic syndrome. Moreover, in men, serum free cortisol levels and cortisol production rates were associated with increased visceral fat and insulin resistance (24).

Most previous studies primarily focused on data from 2020 (15, 16, 25), covering only a limited period even though the COVID-19 pandemic continued until 2021. In contrast, our study analyzed data collected during August to October of both 2020 and 2021 including the period following the start of the vaccination rollout—adding significance to our findings. In particular, Korea’s social distancing policy was implemented through a graded system, where restriction levels were adjusted according to the number of confirmed COVID-19 cases (26). Furthermore, we used data from the Korea Community Health Survey, a nationwide survey, enhancing the generalizability of our results. As noted in previous research (27), social distancing policies may contribute to further obesity in obese populations already affected by obesity. It suggested that consideration for individuals with obesity may have been necessary in the implementation of policies.

This study has several limitations. First of all, as this was a cross-sectional study, it was difficult to establish causal relationships. Although we presented that participants with obesity reported decreased physical activity and increased fast-food intake, it is also possible that those who experienced decreased physical activity and increased fast-food consumption became obese during the COVID-19 period. Second, as obesity and influencing factors were assessed based on a self-reported questionnaire, some discrepancies compared to actual measured values may exist. Participants may have underreported their weight, potentially leading to an underestimation of the obesity prevalence. However, our results are based on relative differences in self-reported decreases in physical activity across BMI categories, rather than actual measurement of BMI. While one study has shown that self-reported BMI did not significantly differ from actual measurements (28), another study showed a tendency toward underreporting BMI, especially among high BMI individuals (29). Self-reported physical activity and dietary habits may have recall bias and social desirability bias (30, 31). A previous study has also shown that women tended to underreport their energy and fat intake (30). Regarding physical activity, self-reported activity levels may differ from objectively measured levels (31). Therefore, the findings of this study may have been underestimated and the overall direction of the results is expected to remain consistent. In addition, this study asked about perceived increases or decreases in activity, and thus the results can be interpreted as reflecting individuals’ subjective evaluations of changes in their behavior. Furthermore, previous research has shown acceptable test–retest reliability for health behavior questionnaires in the KCHS (32), indicating they may be used to assess changes in health behaviors. Third, while physical activity can be influenced by seasonal variation, both the 2020 and 2021 Community Health Surveys were conducted during the same period, from August to October. As such, the likelihood that seasonal effects significantly impacted the reporting of physical activity levels is considered low. Finally, we used quartile-based categorization of daily life restriction scores considering their distribution. This is a commonly used method for categorizing variable but the lack of validation is a limitation of this study. Nevertheless, this study is representative of obese groups by sex and has yielded significant findings, evaluating that obese individuals reported the greater restrictions on daily life.

This study found that individuals with higher obesity levels were more likely to report negative changes in their health behaviors such as decreased physical activity and increased fast food consumption. These findings highlight the need for targeted strategies to support people with obesity during a public health crisis. A recent study showed the clinical effectiveness of telemedicine for the control of chronic diseases including physical conditions (33). A recent study reported that app-based programs for individuals with obesity, including self-management, self-monitoring, and audio- or video-based education, can lead to weight loss (34). Accordingly, various home-based exercise programs, mobile applications for dietary and activity monitoring, and strengthened health education targeted toward high-risk groups might be beneficial for their health. In situations like the COVID-19 pandemic, when face-to-face access is limited, such digital and remote interventions can play a crucial role in supporting the health of vulnerable populations. Accordingly, it may be beneficial for health policies to consider strategies for the management of obesity in these high-risk groups.

## Data Availability

The datasets presented in this study can be found in online repositories. The names of the repository/repositories and accession number(s) can be found below: https://chs.kdca.go.kr/chs/rdr/rdrInfoProcessMain.do.

## References

[1] Yuan Y Jiao B Qu L Yang D Liu R. The development of COVID-19 treatment. Front Immunol. (2023) 14:1125246. doi: 10.3389/fimmu.2023.1125246, PMID: 36776881 PMC9909293

[2] Kim HY Oh IH Lee J Seon JY Jeon WH Park JS . Policy review and modeling analysis of mitigation measures for coronavirus disease epidemic control, health system, and disease burden, South Korea. Emerg Infect Dis. (2021) 27:2753–60. doi: 10.3201/eid2711.203779, PMID: 34429188 PMC8544960

[3] Larsson SC Burgess S. Causal role of high body mass index in multiple chronic diseases: a systematic review and meta-analysis of Mendelian randomization studies. BMC Med. (2021) 19:320. doi: 10.1186/s12916-021-02188-x, PMID: 34906131 PMC8672504

[4] Jeong S-M Jung J-H Yang YS Kim W Cho IY Lee Y-B . 2023 obesity fact sheet: prevalence of obesity and abdominal obesity in adults, adolescents, and children in Korea from 2012 to 2021. JOMES. (2024) 33:27–35. doi: 10.7570/jomes24012 38531533 PMC11000515

[5] Lee M-N Choi Y-S Kim S-D. The leading factors of obesity and severe obesity in Korean adults during the COVID-19 pandemic. Int J Environ Res Public Health. (2022) 19:12214. doi: 10.3390/ijerph191912214, PMID: 36231516 PMC9565112

[6] Cava E Neri B Carbonelli MG Riso S Carbone S. Obesity pandemic during COVID-19 outbreak: narrative review and future considerations. Clin Nutr. (2021) 40:1637–43. doi: 10.1016/j.clnu.2021.02.038, PMID: 33765600 PMC7923945

[7] Restrepo BJ. Obesity prevalence among U.S. adults during the COVID-19 pandemic. Am J Prev Med. (2022) 63:102–6. doi: 10.1016/j.amepre.2022.01.012, PMID: 35725124 PMC8977388

[8] Stockwell S Trott M Tully M Shin J Barnett Y Butler L . Changes in physical activity and sedentary behaviours from before to during the COVID-19 pandemic lockdown: a systematic review. BMJ Open Sport Exercise Med. (2021) 7:e000960. doi: 10.1136/bmjsem-2020-000960 PMC7852071 34192010

[9] Kang YW Ko YS Kim YJ Sung KM Kim HJ Choi HY . Korea community health survey data profiles. Osong Public Health Res Perspect. (2015) 6:211–7. doi: 10.1016/j.phrp.2015.05.003, PMID: 26430619 PMC4551141

[10] Haam J-H Kim BT Kim EM Kwon H Kang J-H Park JH . Diagnosis of obesity: 2022 update of clinical practice guidelines for obesity by the Korean Society for the Study of obesity. J Obes Metab Syndr. (2023) 32:121–9. doi: 10.7570/jomes23031, PMID: 37386771 PMC10327686

[11] Drewnowski A Specter SE. Poverty and obesity: the role of energy density and energy costs. Am J Clin Nutr. (2004) 79:6–16. doi: 10.1093/ajcn/79.1.6, PMID: 14684391

[12] Swinburn BA Sacks G Hall KD McPherson K Finegood DT Moodie ML . The global obesity pandemic: shaped by global drivers and local environments. Lancet. (2011) 378:804–14. doi: 10.1016/S0140-6736(11)60813-1, PMID: 21872749

[13] Jeffery RW Rick AM. Cross-sectional and longitudinal associations between body mass index and marriage-related factors. Obes Res. (2002) 10:809–15. doi: 10.1038/oby.2002.109, PMID: 12181390

[14] Luckhaupt SE Cohen MA Li J Calvert GM. Prevalence of obesity among US workers and associations with occupational factors. Am J Prev Med. (2014) 46:237–48. doi: 10.1016/j.amepre.2013.11.002, PMID: 24512862

[15] Zhu J Di Gessa G Zaninotto P. Changes in health behaviours during the COVID-19 pandemic and effect on weight and obesity among older people in England. Sci Rep. (2023) 13:14661. doi: 10.1038/s41598-023-41391-z, PMID: 37670073 PMC10480155

[16] Garcês CP Oliveirae Silva L Nunes SM Cheik NC. Effects of social distancing caused by the COVID-19 pandemic on physical activity level, sitting time, and binge eating: a comparison between overweight/obese and normal-weight adults. Sport Sci Health. (2022) 18:1505–12. doi: 10.1007/s11332-022-00974-5, PMID: 35818432 PMC9261176

[17] Farrell E Hollmann E Roux CL Nadglowski J McGillicuddy D. At home and at risk: the experiences of Irish adults living with obesity during the COVID-19 pandemic. EClinicalMedicine. (2022) 51:101568. doi: 10.1016/j.eclinm.2022.101568, PMID: 35875819 PMC9289959

[18] Irschik S Brandt JB Eisenkölbl J. COVID-19 pandemic-related weight gain in the pediatric population declined after restrictions ended, except among obese patients. Front Public Health. (2023) 11:1260269. doi: 10.3389/fpubh.2023.1260269, PMID: 37942242 PMC10628554

[19] Yang HJ Park S Yoon T-Y Ryoo J-H Park SK Jung JY . Nationwide changes in physical activity, nutrient intake, and obesity in South Korea during the COVID-19 pandemic era. Front Endocrinol. (2022) 13:965842. doi: 10.3389/fendo.2022.965842, PMID: 36176463 PMC9513223

[20] Kim K Zhang S Ding P Wang Y Yim BH Hu Z . Changes in physical activity and health indicators among Koreans during the COVID-19 pandemic: comparison between 2019 and 2020. Healthcare (Basel). (2022) 10:2549. doi: 10.3390/healthcare10122549 36554071 PMC9777855

[21] Brewis AA Han SY SturtzSreetharan CL. Weight, gender, and depressive symptoms in South Korea. Am J Hum Biol. (2017) 29:e22972. doi: 10.1002/ajhb.22972, PMID: 28161899 PMC5573951

[22] Lee HY Hong BK. Factors related to the weight control practices of Korean adults. JOMES. (2021) 30:365–76. doi: 10.7570/jomes21041, PMID: 34924366 PMC8735822

[23] Epel EE Moyer AE Martin CD Macary S Cummings N Rodin J . Stress-induced cortisol, mood, and fat distribution in men. Obes Res. (1999) 7:9–15. doi: 10.1002/j.1550-8528.1999.tb00385.x, PMID: 10023725

[24] Purnell JQ Kahn SE Samuels MH Brandon D Loriaux DL Brunzell JD. Enhanced cortisol production rates, free cortisol, and 11β-HSD-1 expression correlate with visceral fat and insulin resistance in men: effect of weight loss. Am J Physiol Endocrinol Metab. (2009) 296:E351–7. doi: 10.1152/ajpendo.90769.2008, PMID: 19050176 PMC2645022

[25] Maltoni G Zioutas M Deiana G Biserni GB Pession A Zucchini S. Gender differences in weight gain during lockdown due to COVID-19 pandemic in adolescents with obesity. Nutr Metab Cardiovasc Dis. (2021) 31:2181–5. doi: 10.1016/j.numecd.2021.03.018, PMID: 33994065

[26] Bahk Y-C Jung D Choi K-H. Social distancing policy and mental health during COVID-19 pandemic: an 18-month longitudinal cohort study in South Korea. Front Psychol. (2023) 14:1256240. doi: 10.3389/fpsyg.2023.1256240 37823072 PMC10562579

[27] Almandoz JP Xie L Schellinger JN Mathew MS Gazda C Ofori A . Impact of COVID-19 stay-at-home orders on weight-related behaviours among patients with obesity. Clin Obesity. (2020) 10:e12386. doi: 10.1111/cob.12386, PMID: 32515555 PMC7300461

[28] Hattori A Sturm R. The obesity epidemic and changes in self-report biases in BMI. Obesity. (2013) 21:856–60. doi: 10.1002/oby.20313, PMID: 23712990 PMC5800501

[29] Visscher TL Viet AL Kroesbergen H Seidell JC. Underreporting of BMI in adults and its effect on obesity prevalence estimations in the period 1998 to 2001. Obesity. (2006) 14:2054–63. doi: 10.1038/oby.2006.240, PMID: 17135623

[30] Myers RJ Klesges RC Eck LH Hanson CL Klem ML. Accuracy of self-reports of food intake in obese and normal-weight individuals: effects of obesity on self-reports of dietary intake in adult females. Am J Clin Nutr. (1988) 48:1248–51. doi: 10.1093/ajcn/48.5.1248, PMID: 3189212

[31] Jakicic JM King WC Gibbs BB Rogers RJ Rickman AD Davis KK . Objective versus self-reported physical activity in overweight and obese young adults. J Phys Act Health. (2015) 12:1394–400. doi: 10.1123/jpah.2014-0277, PMID: 25599334 PMC4506910

[32] Kim SJ Han JA Kim YH Choi BY Kim SY Lee HJ . Test-retest reliability of health behavior items in the community health survey in South Korea. Epidemiol Health. (2015) 37:e2015045. doi: 10.4178/epih/e2015045, PMID: 26493776 PMC4722222

[33] de Rezende DRB Neto IA Iunes DH Carvalho LC. Analysis of the effectiveness of remote intervention of patients affected by chronic diseases: a systematic review and meta-analysis. J Med Access. (2023) 7:27550834231197316. doi: 10.1177/27550834231197316, PMID: 37781504 PMC10540568

[34] Gemesi K Winkler S Schmidt-Tesch S Schederecker F Hauner H Holzapfel C. Efficacy of an app-based multimodal lifestyle intervention on body weight in persons with obesity: results from a randomized controlled trial. Int J Obes. (2024) 48:118–26. doi: 10.1038/s41366-023-01415-0, PMID: 38017117 PMC10746538

