# Correction: Obesity, daily life restrictions, and health behaviors during the COVID-19 pandemic in Korea

**DOI:** 10.3389/fpubh.2026.1787112

**Published:** 2026-01-29

**Authors:** Inwook Lee, Yujin Chang, Hye Soon Park, Jung Ah Lee

**Affiliations:** Department of Family Medicine, Asan Medical Center, University of Ulsan College of Medicine, Seoul, Republic of Korea

**Keywords:** COVID-19 pandemic, social distancing, daily life restrictions, obesity, physical activity, fastfood intake

In the published article, there was a mistake in the Abstract where the subheading “Discussion” was incorrectly used; this has been corrected to read “Conclusion.”

In the Discussion section, the term “Australian” was incorrectly used and has been corrected to “Austrian.” A correction has been made to the section **Discussion**, paragraph number 3.

The sentence previously read:

“Additionally, although the study focused on children, a recent study about Australian pediatric population reported that approximately half of overweight children became obese during the pandemic (18).”

A corrected sentence reads:

“Additionally, although the study focused on children, a recent study about Austrian pediatric population reported that approximately half of overweight children became obese during the pandemic (18).”

The original version of this article has been updated.

