# Supplementary material for: Obesity, daily life restrictions, and health behaviors during the COVID-19 pandemic in Korea
Source: Front Public Health. 2025 Oct 31;13:1653576. doi: 10.3389/fpubh.2025.1653576 (PMC12615168; doi:10.3389/fpubh.2025.1653576)
Supplement: Supplementary file 1 [file Table_1.docx]

**Supplementary Table 1. Logistic Regression Analysis of Daily Life Restrictions by Obesity Status with Varying Cutoffs**

|  | **Univariable OR (95% CI)** | **P value** | **P for trend^a^** | **Multivariable OR^b^ (95% CI)** | **P value** | | **P for trend^a^** |
| --- | --- | --- | --- | --- | --- | --- | --- |
| **Men** |  |  |  |  |  |  | |
| **Cut off 10** |  |  | *<0.001* |  |  | *<0.001* | |
| Underweight | 1.06 (0.80-1.41) | 0.695 |  | 0.93 (0.70-1.24) | 0.624 |  | |
| Normal | 1 (reference) |  |  | 1 (reference) |  |  | |
| Obesity I | 1.18 (1.08-1.28) | <0.001 |  | 1.23 (1.13-1.34) | <0.001 |  | |
| Obesity II | 1.28 (1.09-1.49) | 0.002 |  | 1.32 (1.13-1.55) | 0.001 |  | |
| **Cut off 20** |  |  | *<0.001* |  |  | *<0.001* | |
| Underweight | 1.04 (0.85-1.28) | 0.685 |  | 0.93 (0.76-1.15) | 0.512 |  | |
| Normal | 1 (reference) |  |  | 1 (reference) |  |  | |
| Obesity I | 1.14 (1.07-1.21) | <0.001 |  | 1.16 (1.09-1.23) | <0.001 |  | |
| Obesity II | 1.20 (1.07-1.35) | 0.002 |  | 1.19 (1.06-1.34) | 0.004 |  | |
| **Cut off 30** |  |  | *<0.001* |  |  | *<0.001* | |
| Underweight | 1.03 (0.89-1.20) | 0.925 |  | 0.96 (0.82-1.11) | 0.591 |  | |
| Normal | 1 (reference) |  |  | 1 (reference) |  |  | |
| Obesity I | 1.14 (1.09-1.19) | <0.001 |  | 1.14 (1.09-1.20) | <0.001 |  | |
| Obesity II | 1.20 (1.10-1.31) | <0.001 |  | 1.15 (1.05-1.26) | 0.003 |  | |
| **Cut off 50** |  |  | *<0.001* |  |  | *<0.001* | |
| Underweight | 0.99 (0.88-1.11) | 0.858 |  | 0.92 (0.82-1.03) | 0.138 |  | |
| Normal | 1 (reference) |  |  | 1 (reference) |  |  | |
| Obesity I | 1.13 (1.09-1.16) | <0.001 |  | 1.14 (1.10-1.18) | <0.001 |  | |
| Obesity II | 1.12 (1.05-1.21) | 0.001 |  | 1.12 (1.04-1.20) | 0.003 |  | |
| **Women** |  |  |  |  |  |  | |
| **Cut off 10** |  |  | *0.178* |  |  | *0.564* | |
| Underweight | 0.96 (0.83-1.11) | 0.584 |  | 1.02 (0.88-1.17) | 0.829 |  | |
| Normal | 1 (reference) |  |  | 1 (reference) |  |  | |
| Obesity I | 1.01 (0.93-1.11) | 0.758 |  | 0.94 (0.86-1.02) | 0.138 |  | |
| Obesity II | 1.16 (0.96-1.40) | 0.136 |  | 1.09 (0.90-1.32) | 0.404 |  | |
| **Cut off 20** |  |  | *0.136* |  |  | *0.816* | |
| Underweight | 0.96 (0.86-1.06) | 0.386 |  | 0.98 (0.88-1.09) | 0.735 |  | |
| Normal | 1 (reference) |  |  | 1 (reference) |  |  | |
| Obesity I | 1.03 (0.97-1.10) | 0.340 |  | 0.99 (0.93-1.06) | 0.861 |  | |
| Obesity II | 1.05 (0.91-1.21) | 0.495 |  | 1.02 (0.89-1.17) | 0.781 |  | |
| **Cut off 30** |  |  | *0.123* |  |  | *0.271* | |
| Underweight | 1.01 (0.94-1.09) | 0.734 |  | 1.02 (0.95-1.11) | 0.546 |  | |
| Normal | 1 (reference) |  |  | 1 (reference) |  |  | |
| Obesity I | 1.05 (1.00-1.09) | 0.055 |  | 1.04 (0.99-1.09) | 0.113 |  | |
| Obesity II | 1.05 (0.95-1.16) | 0.372 |  | 1.04 (0.94-1.16) | 0.445 |  | |
| **Cut off 50** |  |  | *0.004* |  |  | *0.640* | |
| Underweight | 0.95 (0.89-1.01) | 0.075 |  | 1.00 (0.94-1.06) | 0.887 |  | |
| Normal | 1 (reference) |  |  | 1 (reference) |  |  | |
| Obesity I | 1.04 (1.00-1.08) | 0.030 |  | 1.01 (0.97-1.04) | 0.757 |  | |
| Obesity II | 1.03 (0.95-1.12) | 0.434 |  | 1.01 (0.93-1.10) | 0.744 |  | |

^a^P for trend was calculated based on an ordinal categorical variable of obesity.

^b^Adjusted for age, income, education, marital status, and occupational status.
